# The Benefits and Challenges of the Multimodal Treatment in Advanced/Metastatic Malignant Melanoma

**DOI:** 10.3390/diagnostics13091635

**Published:** 2023-05-05

**Authors:** Alexandru-Rares Stoian, Gabriela Rahnea-Nita, Anda-Natalia Ciuhu, Laurentia Gales, Rodica-Maricela Anghel, Laura-Florentina Rebegea, Roxana-Andreea Rahnea-Nita, Liliana-Florina Andronache, Ioana Soare, Gabriela Stoleriu

**Affiliations:** 1 Clinical Department, Faculty of Medicine, The University of Medicine and Pharmacy “Carol Davila”, 8 Eroii Sanitari Street, 050474 Bucharest, Romania; 2 “Bagdasar-Arseni” Emergency Clinical Hospital, 041915 Bucharest, Romania; 3 “Sf. Luca” Chronic Disease Hospital, 041915 Bucharest, Romania; 4 The Oncological Institute “Prof. Dr. Alexandru Trestioreanu”, 022328 Bucharest, Romania; 5 Radiotherapy Department, “Sf. Ap. Andrei” County Emergency Clinical Hospital, 800579 Galati, Romania; 6 Clinical Department, Faculty of Medicine and Pharmacy, “Dunarea de Jos” University of Galati, 800008 Galati, Romania; 7 Research Center in the Field of Medical and Pharmaceutical Sciences, ReFORM-UDJ, 800010 Galati, Romania; 8 Clinical Department, The Faculty of Medicine, “Titu Maiorescu” University, 040051 Bucharest, Romania

**Keywords:** malignant melanoma, new therapeutic approach, chemotherapy, adverse events

## Abstract

Currently, the treatment of malignant melanoma offers the longest and the most studied experience of innovative treatments in malignant pathology. The algorithm of the therapeutic decision in advanced or metastatic melanoma must comprise: the timing of the therapeutic initiation, the sequencing of the specific oncological treatment (radiotherapy and chemotherapy still being therapeutic alternatives in selected cases), the diagnosis and the management of adverse reactions. We present the case of a patient diagnosed with metastatic malignant melanoma in November 2019, who progressed successively under new systemic treatment throughout the 3 years of treatment and experienced skin reactions of various degrees of severity. The comprehensive response to secondary hilar pulmonary lymphatic determinations under subsequent chemotherapy was specific to the presented case. The occurrence of vitiligo secondary to immunotherapy is a favorable prognostic factor, but the occurrence of secondary cerebral determinations is an extremely severe prognostic factor in malignant melanoma and a challenge in making the therapeutic decision. Previous treatment with immune checkpoint inhibitors may trigger a favorable response to systemic chemotherapy. The early and accurate diagnosis of the adverse events of the new therapies requires a multidisciplinary approach, because it can radically change the therapeutic decision.

## 1. Introduction

Malignant melanoma (MM) has shown an increase in incidence in the last decade, with exposure to ultraviolet radiation as the main triggering factor [1,2,3]. In a study conducted in England, an increase in the incidence of the disease from an average of 837 cases/year between 1981–1985 to 6963 cases/year between 2016–2018 was highlighted [1,2]. On the other hand, in a study carried out in the United States, a rapid decrease in mortality was highlighted through the efficiency of immune and targeted therapies in malignant melanoma [4].

Described since the 14th century B.C.E. (before the Common Era) in the mummies of Peru, malignant melanoma started to be recognized, named and subsequently studied beginning in the 1800s when Rene Laennec introduced the term “melanosis” and Sir Robert Carswell named the malignant pigment lesions of the skin “melanoma” [5,6]. Since 1975, specific systemic therapies for malignant melanoma have been developed, including the approval of Dacarbazine and, subsequently, treatments with high-dose alpha interferon (IFN-α) and high-dose interleukin-2 (IL-2) [6]. Since the 2010s, progress in systemic treatment has become exponential, in response to decades of molecular and immunologic studies of cancers [7,8].

The mechanism of action of Dacarbazine, a standard treatment for malignant melanoma for more than 30 years, has not been elucidated yet [9]. It is considered to have a cytotoxic effect due to its structure as an alkylating agent and because it inhibits deoxyribonucleic acid (DNA) synthesis through its action as a purine analogue [9]. Il-2, through the activation and proliferation of T lymphocytes and the stimulation of natural killer (NK) cells, fostered a real interest in the treatment of cancers at the end of the 1990s, but it demonstrates a response rate of only 15% in metastatic renal cancers and metastatic malignant carcinoma. In high doses, IL-2 has demonstrated a clinical response of up to 50% in the with malignant melanoma with lymphopenia [9]. Type I and II interferons (IFNs) present antitumor action through signal transducer and activator of transcription (STAT) 1 and 2 activations and related mechanisms: the activation of mitogen-activated protein kinase (MAPK) pathway, of mammalian target of rapamycin (mTOR) pathway and of nucleotide Guanosine TriPhosphate (GTP)ases/cyclin-dependent kinases pathway. IFN-α stimulates tumor immunogenicity and the response of antitumor dendritic cells through the polarization, maturation and survival of dendritic cells. Therefore, IFNs have a proapoptotic, anti-proliferative, antiangiogenic and potent immunoregulatory effect [10,11].

Starting from the theory that tumor cells manage to “fool” the immune system through the “peripheral tolerance” mechanism—a process of regulating the activation of T lymphocytes by which autoimmunization is prevented, the treatment of advanced/metastatic malignant melanoma is being revolutionized by immune checkpoint inhibitors. An important role in this process is played by immune control pathways: cytotoxic T-lymphocyte–associated antigen 4 (CTLA-4) and programmed death receptor-1 (PD-1) [12,13]. The CTLA-4 pathway inhibits the autoreactive potential in the initial stage of activation of naïve T cells, at the level of lymph nodes, while the PD-1 pathway regulates this process, at the level of peripheral tissues, in the late phase of the immune process, on the previously activated T cell. PD-1 is expressed both at the level of T lymphocytes and at the level of B lymphocytes, monocytes, dendritic cells and tumor-infiltrating lymphocytes (TILs). By linking to its protein programmed cell death-ligands 1 and 2 (PD-L1 and PD-L2), PD-1 inhibits the activity of T cells [12,13,14]. Tumor cells from malignant melanoma overexpress PD-L1 to escape the inflammatory process. Thus, the treatment of advanced/metastatic malignant melanoma was revolutionized starting in the 2010s through: Ipilimumab (recombinant humanized monoclonal antibody that binds to CTLA-4), Pembrolizumab and Nivolumab (direct anti PD-1 monoclonal antibodies), and Atezolizumab (fully humanized, engineered monoclonal antibody of IgG1 isotype against PD-L1), including the association between anti-CTLA-4 and anti-PD-1 [12,13].

BRAF, named also as NS7; B-raf; BRAF1; RAFB1; B-RAF1; BRAF-1, is a B-Raf proto-oncogene, serine/threonine kinase, the gene that encodes a protein belonging to the RAF family of serine/threonine protein kinases, located on chromosome 7q34. It was noticed that 40–60% of the patients with malignant melanoma present BRAF(h) gene mutations— most frequently, the modification of a single amino acid at the level of codon 600 (BRAFV600E). The oncogenic effect of BRAF mutation occurs by activating the MAPK pathway/extracellular signal-regulated kinase (ERK), named MAPK/ERK kinase or MEK. MEK is activated by MEK phosphorylation and subsequently ERK phosphorylation [15,16,17,18]. This characteristic determines the sensitivity of these tumors to targeted therapies with the inhibition of the MAPK pathway. Thus, BRAF inhibitors (Dabrafenib, Vemurafenib, Encorafenib) demonstrated an objective antitumor response in approximately 50% of the patients with malignant melanoma, but with a short duration of approximately 6 months due to mechanisms of resistance to treatment [15,16,19]. The simultaneous inhibition of BRAF kinase and MEK (trametinib, cobimetinib, binimetinib) determines long-term responses, an increased tumor response rate and a paradoxical decrease in skin toxicity, thus becoming the standard treatment in malignant melanoma with present BRAF mutations, with increases in the disease interval until progression of up to 1.8 months [20].

## 2. Materials and Methods

**Figure 1:**
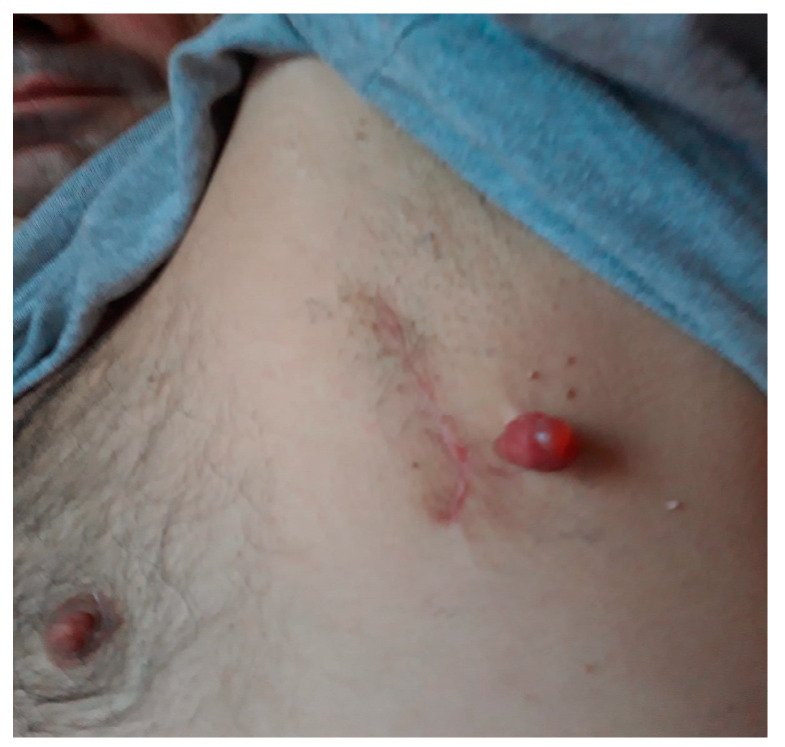
Melanoma relapse, at the time of the patient’s admission to the Surgical Department of our team.

A 52-year-old patient from a rural area, with an occupational history of exposure to sun, presented to our Oncology Department 3 years ago. At that time, the patient had undergone a surgical treatment for a 2/2 cm pigmented ulcerated skin tumor formation located at the level of the left basal posterior chest, 0.5 cm superior to a post-resection scar from a similar skin lesion 12 months earlier (Figure 1).

Histopathological and immunohistochemical tests revealed nodular pigmented malignant melanoma, Breslow 15 mm (local staging pT4b), Clark V, 8 mitoses/mm^2^, in hot-spot, “non-brisk” lymphocytic inflammatory infiltrates, presenting angiotropism, without any lymphatic-vascular invasion, absent neurotropism, with V600E mutation detected in the BRAF gene (c.1799T > A). The pre-therapeutic assessment comprised a computerized tomography (CT) scan of the brain, thorax, abdomen and pelvis with contrast medium, which revealed a thoraco-abdominal aortic aneurysm and lymphoscintigraphy with surgical removal of the left axillary sentinel nodes. A complete left axillary excision was performed, followed by the histopathological and immunohistochemical analysis of the excised sentinel lymph nodes (3 lymph nodes with MM metastases, the largest metastatic mass of 18 mm, subcapsular and extramedullary location, presenting extra lymph node extension, p. V600E mutation in the present BRAF gene), pN2a stage. A positron emission tomography (PET) scan/CT assessment at approximately 1 month after the axillary lymph node removal pointed to a metabolically active upper left perijugular adenopathy with a diameter of 29/30 mm, moderately fixed lymph nodes for 18Fluor-fluorodeoxyglucose (18F-FDG) PET located bilaterally infrahilar and hilar pulmonary, possibly with a non-specific inflammatory substrate; non-capturing pulmonary micronode of 5 mm, medial segment, middle lobe. The subsequent therapeutic decision was established by the multidisciplinary board, taking into account the extremely difficult surgical approach for the metabolically active perijugular lesion and the coronavirus disease 2019 (COVID-19) pandemic at the onset with severe restrictive health regimen, deciding to initiate the systemic oncological treatment. The targeted treatment for the BRAF mutation was initiated with Dabrafenib in the standard dose (150 mg BID (taken two times a day)) and Trametinib 2 mg/day. The patient underwent this treatment for 6 months, with very good clinical tolerance, with a few febrile spikes at the beginning of the treatment that were controlled with antipyretic treatment. The stage reassessment after 6 months, by magnetic resonance imaging (MRI) of the cervical area with contrast medium and the CT of the thorax, abdomen and pelvis with contrast medium, described the progression of the disease at the level of the lymph nodes—a mass with a structure and non-homogeneous gadolinium of 30/22 mm in the axial plane and 44 mm in the craniocaudal diameter, located in the left jugular carotid, with a mass effect on the jugular vein—MRI appearance of adenopathy; multiple bilateral superior latero-cervical adenopathies of 10 mm, submandibular of 13 mm, bilateral deep latero-cervical of 11 mm, bilateral posterior laterocervical of 7 mm, lymph nodes of maximum 22/8 mm in Barety’s space, infracentimeters in the aorto-pulmonary window, lateral-aortic, right hilar of 20/13 mm.

**Figure 2:**
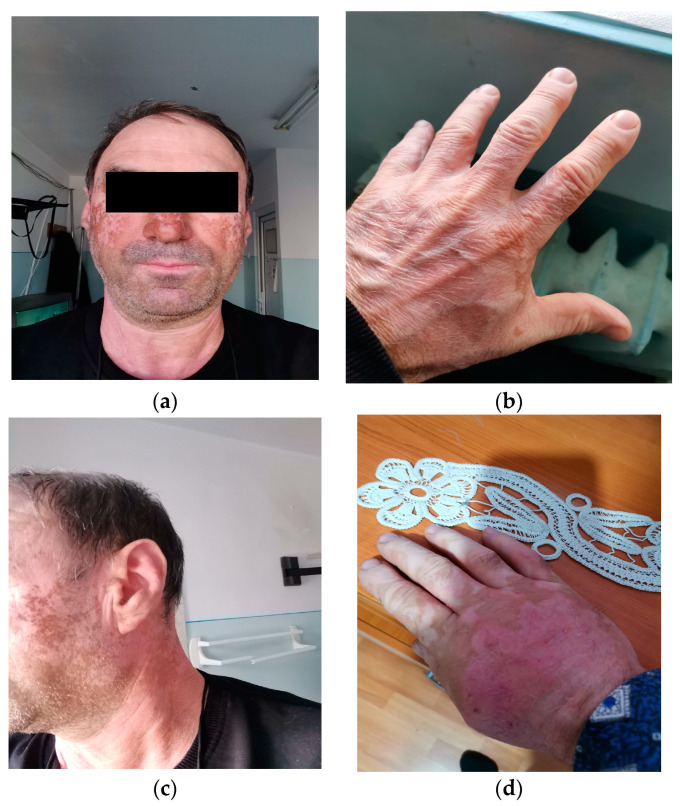
Vitiligo-like lesions in the areas exposed to the sun: (**a**) face and forehead; (**b**) dorsal side of the left hand; (**c**) face, forehead and left side of the neck; (**d**) dorsal side of the right hand.

For the next 10 months, the patient underwent treatment with Nivolumab 240 mg every 14 days, with a temporary interruption of the treatment for 6 weeks due to an increase in serum transaminase values > 5xN, until the correction of the values under corticotherapy. The occurrence of skin depigmentation was also noticed in the last 3 months of nivolumab administration, predominantly at the level of the face and bilateral palms with a clinical aspect of “vitiligo” (Figure 2), and at the level of the distal lateral extremity of the postoperative scar, a 5 mm brown-blue lesion with a clinical aspect of malignant melanoma relapse.

**Figure 3:**
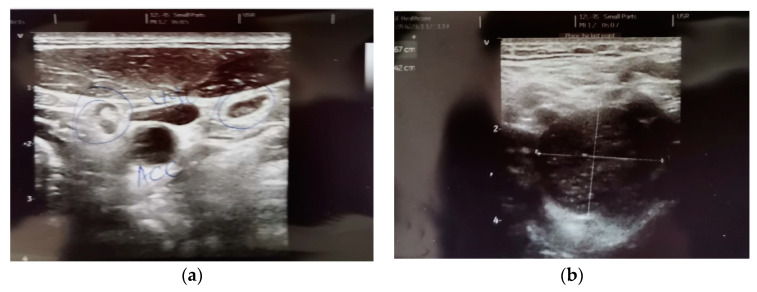
(**a**) Two nodular images, with the hyperechoic center of 0.66 and, respectively, 0.53 cm, between external to common carotid artery (CCA) and internal jugular vein (IJV). (**b**) Hypoechoic nodular image, with Doppler signal in the hilum, the transverse diameter of 2.57/2.42 cm; longitudinal diameter of 4.54/2.14 cm, between internal carotid artery (ICA) and external carotid artery (ECA), in direct contact with the vessels.—April 2021.

After 10 months of treatment, the imaging evaluations, the CT scan of the cervical, thoracic and abdominal area with contrast medium, the ultrasound of soft tissues in the left lateral cervical area (Figure 3), and the angiography and the MRI of the cervical area with contrast medium revealed the increase in the tumor mass located in the left jugular carotid area, with no progression of the other lymph node lesions described in previous examinations (an image with adenopathy including necrotic areas, medial to the left jugular carotid vascular mass of 58.7/26/24 mm, with a structure similar to adenomegalies upon MRI examination). The patient was re-evaluated by the therapeutic indication board, and a surgical approach was recommended for the lesions in progression. The histopathological and immunohistochemical tests of the tumor lesion in the left cervical area revealed Sry-related HMg-Box gene 10 (S100 SOX10) intensely positive in the tumor proliferation, epithelial membrane antigen (EMA)—intensely positive at the level of the fibrous capsule, Ki67 < 5%—Schwannoma, and for the previously described skin formation—malignant melanoma relapse. The patient continued the treatment with Nivolumab, in the same therapeutic sequence for 4 more months, until the next PET/CT imaging evaluation when the metabolic and dimensional progression of a left lung adenopathy up to 22/11 mm and standardized uptake values (SUV) of 10.13 were revealed. The histopathological and immunohistochemical assessment of the lymph node lesion confirmed the progression of the disease under treatment through malignant melanoma metastasis with the presence of BRAF mutation.

After using the combination of BRAF + MEK inhibitors as the first line and treatment, with immune checkpoint inhibitors as a second line of treatment, i.e., the anti PD-1, treatment was limited to chemotherapy, anti CTLA-4 (ipilimumab), and radiotherapy (controversial)/re-challenge of the treatment with Dabrafenib + Trametinib (the BRAF + MEK inhibitor combination in Romania). In January 2022, the targeted treatment was reinitiated, keeping the standard doses of treatment—Dabrafenib 150 mg BID (taken two times a day), Trametinib 2 mg/day, but with extremely difficult clinical tolerance, manifested by daily chills, facial edema, including that of the lips, generalized pustular erythematous, pruriginous eruption, with areas of integumentary peeling and depigmentation—symptoms that are not controlled by corticoid and antihistaminic treatment. After a prolonged discontinuation of treatment for approximately 6 weeks, until the disappearance of the clinical complaints, the patient required admission to the Oncology Department for ongoing hospitalization for the titration of systemic treatment and clinical monitoring of the symptoms. The clinical oncological and dermatological evaluation upon the attempt to resume the treatment with Dabrafenib and Trametinib guided the diagnosis to allergic reactions to the treatment but could not rule out a cutaneous adverse reaction with severely increased potential.

Due to the progression of the disease in the intrathoracic ganglionic areas, especially at the level of the right pulmonary hilum, the treatment with Dacarbazine 500 mg/m^2^ every 21 days was initiated from April 2022 until September 2022, when the patient exhibited partial motor epileptic seizures on the right side of the body, motor deficit of the right (crural > brachial) hemibody, dysphasia and headache. The patient’s emergency assessment within the Neurosurgical Department revealed a left parasagittal frontal-parietal cerebral tumor with a cystic component, of 36/50/44 mm in dimension, subsequently completely resected. Histopathological and immunochemical tests made the diagnosis of amelanotic malignant melanoma metastasis. Treatment with external stereotactic irradiation of the target volumes was completed, with total dose (TD) of 25 Gy left frontal-parietal tumor bed, using intensity-modulated radiation therapy and volumetric modulated arc therapy (IMRT-VMAT) technique, respectively, with TD 30 Gy for tumor relapse of 7 mm, revealed upon cerebral MRI assessment with contrast medium. It is worth mentioning that the thoracic imaging evaluation described the complete response to the treatment with Dacarbazine at the level of the intrathoracic lymph node areas.

Three weeks after completing radiotherapy, the patient presented again as an emergency case to the Neurosurgical Department with severe neurologic symptoms for a significant cerebral edema with thrombosis in the sagittal sinus and continuation in the evolution of the cerebral disease through secondary leptomeningeal determinations. The patient ended his struggle with the disease on Christmas day, after 2 months of “best supportive care”.

## 3. Discussion

We consider that the peculiarities of our case are as follows:

- The description, in all of the imaging investigations carried out initially, but also in the reassessment of the treatment, of a left latero-cervical adenopathy, stages II-III, with dimensional progression under treatment with BRAF/MEK inhibitors and Nivolumab—histopathologically and immunohistochemically invalidated during the surgical verification—schwannoma;
- The development of a “vitiligo”-like cutaneous lesion with evolution at a distance, during the treatment with Nivolumab;
- A 3rd degree severity skin reaction when BRAF/MEK inhibitors were reintroduced into the treatment—an allergic reaction to treatment vs. an adverse skin reaction;
- An imaging response of intrathoracic ganglia to the treatment with Dacarbazine, after the progression of these lesions under targeted treatment and immune checkpoint inhibitors;
- Throughout the evolution of the disease, a single cerebral secondary determination occurred, thus challenging the therapeutic decision.

Considering the clinical and imaging diagnosis of schwannomas at the level of the head and neck, the specialized literature only mentions one incidence of this pathology of 5% of all soft tissue tumors, 45% being diagnosed at the peripheral or autonomic nerve level of the head and neck, with symptoms associated with the nerve involved in the tumor transformation [21,22]. Most frequently, schwannoma is associated with type II neurofibromatosis, more rarely with type I, and it rarely becomes malignant [21]. It presents a slow increase in dimensions, and it is described either as a small mass, homogenous or more frequently well circumscribed tumors, heterogenous, iso/hypodense compared to muscular structures, with cystic formations inside and with the displacement of the adjacent structures upon the imaging evaluation [21,23]. The FDG-PET/CT scan revealed that benign schwannomas present a varied behavior, being able to present increased metabolic activity, acting like a malignant tumor [24]. The imaging descriptions, in our case, either ultrasound, computed tomography or magnetic resonance imaging, led to the diagnosis of an adenopathic image, indeed with a heterogeneous appearance of the tumor mass and with the displacement of adjacent structures, without their invasion and with an increase in dimensions of approximately 1.8 cm in 6 months, with increased metabolic activity at 18F-FDG-PET/CT, which determined the change of the systemic treatment, from a targeted treatment for the BRAF mutation to immune checkpoint inhibitors—Nivolumab, until the pathological examination.

The treatment with immune checkpoint inhibitors (anti-CTLA-4, anti-PD-1, anti-PD-L1) caused a prolonged T-lymphocyte activation [25]. This hyperactivation of CD8+ T-lymphocytes seems to be the mechanism that determines the occurrence of vitiligo-like skin lesions in patients with malignant melanoma under treatment with immune checkpoint inhibitors, called “vitiligo-like depigmentation” [25,26]. The incidence of occurrence is 10–28% among patients with malignant melanoma, with an average onset period of 9 months from the initiation of treatment, and more frequently described in patients under treatment with Pembrolizumab or Nivolumab [25,26]. Skin depigmentation was described in elderly patients and most frequently located in the areas exposed to the sun: the face, the neck region, the cleavage, and the palms, and it is described as a positive predictive factor for the response and survival rate. A decrease of 40–50% in the risk of disease progression was described [26]. We interpret the occurrence of vitiligo-like skin depigmentation in our patient 4 months after the initiation of treatment with Nivolumab as an adverse reaction to the treatment, with the expectation of a sustained and prolonged response to the administration of anti-PD-1, but the progression of the disease was initially noted after 6 months of treatment and confirmed after another 3 months of therapy.

After the progression of the disease at the level of the mediastinal lymph nodes, confirmed by imaging scans and lymph node biopsy, the option was to “re-challenge” the treatment with BRAF/MEK inhibitors [27,28]—once, due to the “false progression” established in the first therapeutic line (through the progression of a lesion that turned out to be schwannoma), the second time due to the scientific synthesis that reported a disease control rate of 57%, a complete response in 8% of the cases, partial response in 20% of the cases and stable disease in 28% of the cases [27,28]. If, upon the first administration of BRAF/MEK inhibitors, the clinical tolerance of the treatment was a very good one, with minimal adverse reactions, at the second administration, we faced skin reactions from the very beginning with extension of medium severity at the level of the jugular mucosa, difficult to manage due to the fact that the patient followed the treatment at home in another city, and he required the interruption of treatment and the initiation of treatment with corticoids and antihistaminic drugs at home, through recommendation by phone. Therefore, the severity of the skin reactions could only be quantified through the patient’s case history and through the remaining skin lesions clinically evaluated upon presentation. The following are known as severe adverse skin reactions to the administration of BRAF/MEK inhibitors: Stevens–Johnson syndrome and toxic epidermal necrolysis, with a lethal potential in evolution [29]. The mean onset period of these skin manifestations was reported to be between 11 and 15 days from the beginning of the treatment [29]. The most potent agent in the occurrence of these adverse reactions proved to be the treatment with Vemurafenib, but they were also reported during the treatment with Dabrafenib [29]. During the evaluation by phone of the adverse reactions experienced by the patient at home, a potentially severe evolution was suspected toward the Stevens–Johnson syndrome, and the immediate interruption of the treatment was indicated along with the initiation of the corticoid treatment. The clinical and biological evaluation (specific markers for immediate or delayed allergic reactions) with the titration of the treatment under strict medical control delayed the therapeutic decision by approximately 4 weeks, and it was not possible to clearly differentiate between the two probabilities that could induce the described skin reactions. This had an impact on the subsequent therapeutic decisions, of delaying the treatment with Ipilimumab and initiating the treatment with Dacarbazine.

Around 40–50% of the patients with malignant melanoma develop cerebral metastases along the evolution of the disease, exhibiting high cerebral tropism after lung and breast cancers [30,31]. The occurrence of cerebral metastases represents a poor prognostic factor for the evolution of the disease, also influenced by the site of metastasis, the number of metastases and their size [30,31]. In the case of oligometastases, surgical treatment remains the first therapeutic option. However, radiotherapy and the new systemic treatments bring real benefits in disease control and survival rates. Stereotactic radiosurgery has added a benefit of 8–10 months to the mean survival period [32]. The combination of BRAF/MEK inhibitors determines greater responses than 58% in intracranial disease, but with short duration of response, and the treatment with anti-PD-1 demonstrated durable responses in 20% of the cases [30,31,32]. Ipilimumab and the combination of Ipilimumab with anti-PD-1 seem to have the most sustained benefit in the systemic treatment of brain metastases in patients with malignant melanoma so far [32]. However, the association of the surgical treatment with stereotactic radiosurgery, followed by the administration of systemic treatment, has been proven to provide the best benefit to survival [32]. Therefore, in the case of a young patient with very good performance status until the onset of the neurological symptoms, with proof of a single cerebral metastasis, the therapeutic approach was the neurosurgical treatment, followed by stereotactic radiotherapy. The complete imaging response of the disease to the systemic treatment with Dacarbazine did not show any benefit in the control of the intracranial disease, for which reason, after the local control of the cerebral disease, the patient’s case was to be re-analyzed by the oncology indication board in order to establish the opportunity for the treatment with anti-CTLA-4 and the risk of immune-mediated adverse skin reactions with lethal potential.

The most frequent and severe complications of stereotactic radiotherapy are radionecrosis and hemorrhage. Radionecrosis occurs in 50% of the patients, with complications described in 30% of the patients, and exhibits benefits after the treatment with corticoids [32,33]. Unfortunately, our patient also presented extensive thrombosis of the sagittal sinus associated with the early radionecrosis after stereotactic radiotherapy, with unfavorable evolution under corticotherapy and low molecular weight heparin in the therapeutic dose, with death occurring as a result of these complications.

In an analysis of 463 patients with malignant melanoma who underwent chemotherapy after anti-PD-1 treatment, a number of 0.4% for complete responses were reported in a medial progression-free interval of 2.5 months [34]. The best response in terms of the objective response rate and the free interval until the progression of the disease was identified in the case of the treatment with Taxane. Studies on “rescue” chemotherapy after the administration of immune checkpoint inhibitors in other cancer sites have reported a possible benefit of this therapeutic sequence [34,35,36].

The innovative treatments of the past 20 years have brought clear benefits to the treatment of advanced/metastatic malignant melanoma:

- Highly increased clinical effectiveness;
- Combined therapies (e.g., BRAF/MEK inhibitors) that increase clinical tolerance and decrease toxicity;
- Active interventions in advanced disease in order to increase the disease-free interval with the approval of new therapies for adjuvant purposes (e.g., Nivolumab, Pembrolizumab, Dabrafenib/Trametinib);
- Therapeutic diversity with the possibility of disease attack, in case of progression under a therapeutic option, in patients with good performance status;
- Increased survival until disease progression, increased overall survival, lasting benefit of the treatment.

However, there are also new challenges:

- The development of new algorithms in the sequential administration of these therapeutic agents;
- The establishment of the best interval of the disease for the initiation of the specific treatment—adjuvant/“follow-up”;
- The multidisciplinary approach to the disease—in establishing the sequence of the specific oncological treatments—surgery, systemic treatment, radiotherapy;
- The selection of patients for whom certain treatments bring maximum benefit;
- The early and correct diagnosis and the control of the adverse reactions of the new therapies by the multidisciplinary team.

## 4. Conclusions

Despite the current systemic therapeutic diversity, the choice of the therapeutic sequence (surgery/systemic treatment/radiotherapy) remains the first important decision-making step.

Any suspicious lesion (especially in the case of oligometastatic neoplasms) must be histopathologically examined.

In the context of the description, it is necessary to augment the interdisciplinary team with specialists in dermatology and allergy for the new therapeutic variants of skin reactions with severe evolutionary potential (e.g., drug-induced hypersensitivity syndrome, Stevens–Johnson syndrome and toxic epidermic necrolysis).

The complications of current treatments with severe potential must be evaluated, diagnosed and treated early to ensure the continuity of the oncological treatment.

After treatment with BRAF/MEK inhibitors or immune checkpoint inhibitors, systemic chemotherapy (e.g., Dacarbazine) can still have benefits in the treatment of metastatic melanoma.

## Data Availability

The data presented in this study are available on reasonable request from the corresponding authors.
